# Social Networks and Health: Understanding the Nuances of Healthcare Access between Urban and Rural Populations

**DOI:** 10.3390/ijerph15050973

**Published:** 2018-05-13

**Authors:** Padmore Adusei Amoah, Joseph Edusei, David Amuzu

**Affiliations:** 1 Division of Graduate Studies and Asia Pacific Institute of Ageing Studies, Lingnan University, Hong Kong, China; 2 Centre for Settlements Studies, Kwame Nkrumah University of Science and Technology, PMB, Kumasi, Ghana; joeedus@yahoo.com; 3 Department of Geography and Sustainability, University of Lausanne, 1015 Lausanne, Switzerland; amuzudave@gmail.com

**Keywords:** social networks, access to healthcare, trust, health, rural and urban, Ghana

## Abstract

Communities and individuals in many sub-Saharan African countries often face limited access to healthcare. Hence, many rely on social networks to enhance their chances for adequate health care. While this knowledge is well-established, little is known about the nuances of how different population groups activate these networks to improve access to healthcare. This paper examines how rural and urban dwellers in the Ashanti Region in Ghana distinctively and systematically activate their social networks to enhance access to healthcare. It uses a qualitative cross-sectional design, with in-depth interviews of 79 primary participants (28 urban and 51 rural residents) in addition to the views of eight community leaders and eight health personnel. It was discovered that both intimate and distanced social networks for healthcare are activated at different periods by rural and urban residents. Four main stages of social networks activation, comprising different individuals and groups were observed among rural and urban dwellers. Among both groups, physical proximity, privacy, trust and sense of fairness, socio-cultural meaning attached to health problems, and perceived knowledge and other resources (mainly money) held in specific networks inherently influenced social network activation. The paper posits that a critical analysis of social networks may help to tailor policy contents to individuals and groups with limited access to healthcare.

## 1. Introduction

Existing studies have demonstrated how classic health determinants such as personal behaviours, natural environment, genetic and social factors distinctively affect health [1,2,3]. Nonetheless, little is known about how these factors interact to shape health and well-being [4]. In part, this paper addresses this lacuna by exploring how rural and urban residents in Ashanti Region in Ghana rely on various forms of social networks to access healthcare. There are alternative views which indicate that access to healthcare is not a critical determinant of health [5]. However, recent research shows that adequate access to healthcare promotes and plays a crucial role in curing and managing dire health problems [5,6,7]. For instance, previous estimates suggest that between 41–72% of under-five mortality in many sub-Saharan African countries could be avoided through sufficient access to healthcare [8]. 

Despite several interventions, important barriers to adequate access to healthcare persist in Ghana [9,10]. Low access to healthcare is mostly attributed to financial barriers and insufficient general and specialised health facilities/services [9,10]. Other impediments comprise religious and culturally-driven preferences, and organisational and procedural constraints [11,12]. Importantly, the conditions and extent of healthcare access, as well as the associated barriers, are different for rural and urban dwellers [9,13]. Studies show that rural residents are comparatively disadvantaged in both quantity and quality of health services as well as knowledge about prevailing health systems [9]. Research suggests that for urban dwellers, critical demographic characteristics such as gender, education and age put some groups at a disadvantage amid increasing urban poverty [14,15,16]. In Ghana, settlers in both rural and urban enclaves face financial barriers to healthcare [9,11,17]. It was anticipated that the introduction of the National Health Insurance Scheme (NHIS) in Ghana would protect against financial difficulties in access to healthcare. Nevertheless, many households are unable or decline to subscribe due to several internal and extraneous (e.g. individual beliefs and preferences) challenges burdening the scheme [9,18,19].

In the wake of such hindrances (financial barriers, inadequate facilities, socio-cultural beliefs), social networks have become a compelling alternative for accessing healthcare by curtailing potential challenges. Social networks refer to “connections among people, organisations, political entities (states or nations), and other units” [20]. They are thus generated through interactions such as talking to others within a community or an organisation [20]. Many people depend on these networks for not only material resources but also knowledge and information to make decisions regarding healthcare access [21,22]. Social networks exude resources—social capital—which both poor and affluent can deploy in times of crisis such as health afflictions [23]. Although the results are inconsistent, there are significant indications that social networks alter demand for health services. This is often through its influence on perceived efficacy or desirability of available services [12,24,25]. For instance, relationships with close social acquaintances such as family members and friends are usually sources of financial, informational and emotional support for the poor and vulnerable [12,24,26,27,28]. Moreover, research in both high and low-income countries such as Ghana has shown that connections with people in the upper part of a social hierarchy or more resourceful persons (such as community leaders and health personnel) are positively related to use of health services through information provision and the offering of social credits [17,25,29].

Furthermore, weak networks tend to provide unique resources (particularly information), which a person may not locate within their usual and close social circles. Weak networks consist of relationships with others who are not related and have dissimilar socio-economic characteristics. It often includes neighbours, a friend of one’s close friend, people with distinct ethnicities, and networks through groups [26,30,31,32]. Extant evidence also suggests that the extent to which any category or type of social network affects health and decision to use specific health services are predicated on abstract forms of social networks such as trust, fairness and reciprocity. These elements are thus sometimes known as the forces that make social networks function and define the degree of intimacy [33,34]. Moreover, given the increase in access to the internet and mobile technologies, the role of social networks and how they are activated are less dependent on proximity as virtual interactions continue to rise [35]. Thus, the nature and conceptualisation of social networks of a person can be complicated and at multiple levels including micro level (individual), macro-level (community levels) and meso level (the interactions between individuals and community level factors) [35,36]. 

Despite such extensive evidence on the influence of social networks on access to healthcare, little is known about the nuances of how, when and why people seek assistance from specific group, institutions or individuals [37]. Inspired by the social organisation strategy (SOS) framework [38], this study disentangles how rural and urban residents make use of their social networks before they finally get in contact with the health system—either formal orthodox or registered traditional services. It focuses on the structure of the social networks of contacts. The SOS framework argues for a socially constructed pattern of decision-making including consultations with others. It posits that social interactions are the basis of social life, and social networks provide the mechanism through which individuals learn about, come to understand and attempt to handle difficulties. Thus, the SOS framework somewhat contradicts the rational choice theory—a theory based on principles of economic psychology [38]. Rational choice theory views individuals as rational actors who engage in purposive actions by calculating the likely costs and benefits of all actions before making a decision [39]. The SOS frameworks instead posit that human choices are social network and event centred [38]. 

In part, this approach, as adopted by the present study, posits that the ‘self’ or individuals and the decisions they make about livelihood problems are products of social interactions. The SOS framework presents the process of decision making by individuals as an episodic one, where the decisions and actions as well as the nature of the social networks (who is involved and in what capacity) can change at any stage given prevailing conditions. The preference formation, the situation and processes involved in decision making are all embedded in the social process according to the SOS framework. In the process, choices to address existing and emergent problems are constructed. However, the nature of a problem at hand determines when, and which aspect of one’s latent social relationships are activated [38,40,41]. The process of constructing a network given a problem can shift the trajectory and dynamics of social life by continuously including or excluding some networks in subtle and profound ways. This theoretical overview is linked to medical decision making regarding how individuals consider their social networks, given the prevailing norms and social values to decide on how to address a health problem [38]. The nature of the process in terms of structure and content can delay or fast-track the process of receiving needed care and affect the efficacy of care that is received [21,42].

## 2. Methods

### 2.1. Study Design

The article is based on a cross-sectional qualitative design. It adopts an interpretivist epistemology with a focus on abductive reasoning to portray the lived experiences and choices of the participants [43,44,45]. Interpretivism helps to deconstruct reality through intra-subjective and intersubjective meaning and understanding of the social world [45]. Its inherent advocation for contextual attachment makes it apposite for this study considering the interest in rural and urban livelihoods concerning access to healthcare. Furthermore, the abductive reasoning approach extends the concentration on the worldview of the participants to consider the social scientific account of the social world as observed from the participants’ perspective while not losing sight of the researched [43]. The study ensured trustworthiness of the process through substantive and ethical validation [45]. By this approach, we endeavoured to provide thorough and comprehensive evidence of our understanding of the research objective and processes, which informed our interpretations through constant reflexivity by opening minds to alternative meanings. 

The Committee on Human Research Publication, and Ethics (CHRPE) of School of Medical Sciences, Kwame Nkrumah University of Science and Technology and Okomfo Anokye Teaching Hospital, Kumasi, Ghana, (CHRPE/AP/345/15) approved the study. Informed consent was obtained from all participants. Moreover, all the names used in the study are pseudonyms that were constructed together with the participants before the interviews to ensure anonymity. 

### 2.2. Data Collection

Given the broader concern on matters of healthcare access, the study purposively gathered data from 79 primary participants using a semi-structured interview guide in a multi-stage cluster sampling [44]. The process consisted of segmentation of urban areas into strata using distinctive characteristics such as economic variations, and ethnicity and religious composition of a locality. Classification of a place as rural or urban was based on the official definition by the Ghana Statistical Service (GSS) in addition to economic characteristics and conditions of social services in a vicinity [46]. Other characteristics considered comprised age, educational attainment, and gender to ensure a balanced demographic. The study only considered adults—persons 18 years and older. This group was more likely to have engaged with the health system directly or even indirectly. Twenty-eight urban and 51 rural residents took part in the study. The difference in sample sizes for both groups were as a result of the adoption of the concept of theoretical saturation in the data gathering process [44]. The participants included both males (47%) and females (53%). Most of them were aged 25 to 44 years in both residential groups. There were approximately 18% and 15% of participants aged 15–24 years in urban and rural localities respectively. As many as 14% and 13% of urban and rural dwellers (respectively) were aged 60 years and above. Junior High School was the typical highest educational attainment for both urban and rural participants. Also, eight community leaders and eight health personnel (including nurses, physicians and pharmacists), added to the accounts of the primary participants through semi-structured in-depth interviews. The participants were selected from eight rural communities (from three districts, namely Atwima Kwanwoma District, Ejisu Juaben Municipality, and Kwabre East District). The urban areas consisted of 36 urban communities/suburbs selected from Kumasi Metropolitan Area and Asokore-Mampong Municipal Area. The data was gathered from June 2015 to October 2015.

The in-depth discussions with the participants (individually) typically consisted of general questions on accessibility to healthcare regarding affordability, availability of services, organisation of the services and geographical access. The discussions later extended to how various social networks of participants contribute to access to healthcare through conscious activation by the participants. All the participants were asked to systematically and hierarchically elaborate (in terms of importance), their decision-making process and the actions they took before finally engaging with the formal health system in both critical and uncritical situations. Emphasis was placed on the individuals, groups and organisations they consulted in those decisions and the actions and reasons behind them. The form of support they sought or received from such persons could be in the form of instrumental help (e.g., money and doing chores), emotional support or information. As argued earlier, this mimics what Knoke [47] refers to as activated ties—the list of persons, organizations/groups—that individuals actually contact in the face of [health-related] adversities. We concentrated on activated form of network approach (i.e., the one a participant had previously used) to elicit the list of persons/groups that the participants (individuals) consulted in the face of their health problems. Research shows that problems with social network recall are usually minimal even among persons with mental problems as matters of health are often eventful [36]. All the interviews were carried out by the first author and two trained research assistants using the same interview guide. The interviews were primarily conducted in ‘Twi’—the predominant local language. It was observed in the pretesting phase of the study that many participants could not express themselves fluently in English. This informed the use of ‘Twi’. All the interviews were taped (audio) with permission from participants. The interviews lasted between 35 to 60 min. 

### 2.3. Data Analysis

The analysis consisted of thematic and narrative approaches [43,44]. A combination of these two methods helped to test and generate a conceptual understanding of the role of social networks in access to healthcare. The analysis began during the field study through reflexive note taking and daily debriefing of the research team. The interview tapes were transcribed verbatim by the research team. However, to ensure the credibility of the process, one local language expert helped to validate the transcripts. The transcripts were coded using open coding method. Through this process, we were able to categorise various social groups and individuals who regularly featured in the social worlds of the participants for access to healthcare. Each of the codes was given a specific identifiable everyday name that made them distinct from others. For each participant, the names were mapped systematically beginning with the frequent point of contact to the last. The processes ended with a collation and juxtaposition of codes for participants in each category (rural or urban dwellers). Through the abductive reasoning approach, the research team framed a conceptual representation of social network participation in decision making about healthcare for both groups. The narrative approach was used to elicit and present stories about the experiences of participants on how they previously activated different aspects of their social networks for healthcare access. The product of the analysis was subjected to scrutiny by one social health researcher. Further to that, community and opinion leaders in the study areas assisted, in a debriefing session, to validate the findings. Each of the authors also presented their independent critique of the process. 

## 3. Findings

The findings are grouped under two broad headings, namely the dependence on social networks for health matters and, the process of social network activation. They are subsequently presented in two heuristic frameworks, which depict different social networks and when they were activated among rural and urban people. 

### 3.1. Dependence on Social Connections for Decisions on Access to Healthcare

Reliance on different forms of social networks on matters of healthcare access was a common phenomenon. In fact, the concerns expressed by participants alluded to the fact that in both rural and urban areas, people relied on their social networks—especially close relatives and friends—to contribute towards improving their chances for healthcare. The primary area of concern was related to healthcare financing as some participants and health personnel revealed:

If I were financially sound I’d visit the hospital regularly as I’ve not been feeling healthy lately. ...Last time I called one of my children who lives at Accra to inform him about my expired health insurance, and he promised to send me some money. One of them here in Kumasi has also promised to send me some money. ...They’ve not delivered on their promises, but I’m waiting patiently.

*(Akua, 57 years Suame, urban)*

…People here are very poor. For instance, one girl went into labour here (CHPS compound) whom I graded as a pauper, so the labour cost was pre-financed by the clinic. I wanted to transfer her to Ejisu Hospital [District hospital] but owing to her financial situation; I had to keep her here. … Later, I called her boyfriend to settle the bills. …He came [with some of his friends], but he did not have any money. …He sought permission to make payment later in the day. …Interestingly, his friends went around from house to house to seek help from their peers to settle the debt. …They were able to raise part of the money. They raised GH¢100 [US$ 25.6] out of a total debt of GH¢180 [US $ 46.2]”.

*(Medical officer 1, rural)*

Some counted on their networks to supplement efforts geared at accessing healthcare. In the absence of any assistance, the act of seeking medical care was a painful experience, as some people had to walk long distances to save money for direct care expenses:

“My grandchildren take care of me. …They feed me and help me do everything at home but they are all jobless. …All my children have died except for only one who does not live here. Right now, I do not feel very well but I do not have money even for transportation to the hospital. I cannot walk all the way to Abira (the nearest community with a health centre).

*(Ama, 70 years, Krobo, rural)*

Moreover, in seeking healthcare, many also consciously relied on their weak ties including area neighbours and distanced friends, to make decisions about the type and location of facilities to seek care. In many such instances, the objective was to look for quality but also the cheapest place to seek care. In the process, the previous experiences of others constituted the yardstick in making the final choice as the experiences of some participants revealed:

My neighbour recommended the clinic which I usually attend to me. …She told me that the doctor at the clinic (Dida health centre) is very good and the healthcare charges are moderate as compared to Foase hospital (District government hospital). …I tried it, and it was true. I’ve since been going there ….

*(Akam, 26 years, Afrancho, rural)*

Such snowball approach to selecting health facilities was also practised among urban residents as some participants confessed to encouraging others to access particular health facilities based on their own experiences. Moreover, rural residents in particular also relied on the benevolence of their religious associations to enhance healthcare access:

…I depend on my church for money. ...The church leaders visit me every month and they always give me GH¢ 10 for upkeep …I use it mostly to buy my drugs or pay for my transportation for my check-up [About US$ 2.6]”.

*(Koo, 51 years, Apem, rural)*

Informational support sometimes accompanied such benevolence. Many relied on these social groups to make specific and general decisions about their healthcare. The significance of the social networks in healthcare access was vividly expressed in how some urban participants bitterly complained about the inactions of their social circles to lend emotional support in times of ill health:

I fell sick not long ago. I was admitted at Komfo Anokye (A teaching hospital) for a week…. Do you know that none of my housemates and even my sisters didn’t visit me? …They are all here in Kumasi. I’ve learned a great lesson. I’ll not concern myself with anyone’s issue anymore.

*(Piaw, 42 years, Kwadaso, urban)*

### 3.2. The Process of Social Network Activation

**Figure 1:**
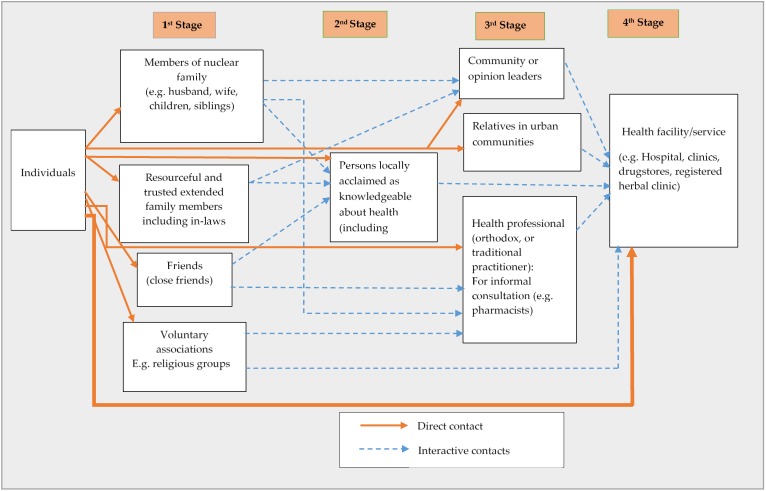
The process of social network activation for access to healthcare among rural inhabitants.

**Figure 2:**
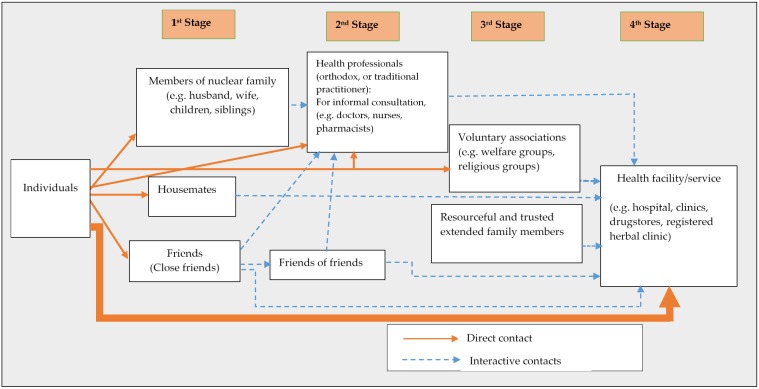
The process of social network activation for access to healthcare among urban residents.

Further to the participants’ elaboration on how different social networks contributed to healthcare access, they were led to portray the mechanism involved in activating their social networks. These experiences of participants are presented textually and graphically depicted through abductive reasoning. Among both urban and rural residents, four main stages, comprising different forms of social networks were unearthed as shown in Figure 1 and Figure 2.

(a) Rural settings:

It emerged among rural inhabitants that health-related issues were first discussed within the family—both nuclear and extended ones. However, even at this level, some cognitive elements such as trust, knowledge and experiences of people were considered before a decision was made. This was demonstrated in how one participant preferred to discuss health issues with his brother and a friend rather than his wife:

For me, I’ve a junior brother and a close friend whom I share these stuff (Health-related matters) with. My brother previously worked at the Komfo Anokye Hospital as a security man, so he knows much about health issues. …My friend also has some knowledge about health matters, and he gives me advice on health issues. After them, the next person is my wife. …If my condition becomes so severe that I need someone to take me to the hospital, then I ask my wife to inform my junior brother for help.

*(Ose, 42 years, Apemanim, rural)*

Members of one’s extended family were easy to locate as the majority of residents in rural settings were indigenes. In fact, many houses accommodated both extended and nuclear families of the same lineage. Additionally, in rural areas, members of religious groups were among the first point of call for people in single households—especially older persons. Intimate friends were a vital source of health information and related directives. Moreover, it was apparent that the need for privacy, and again, trust, played a crucial role in determining who and which groups were first consulted:

…I usually inform my friend first. He knows a lot about health issues. …Also, I don’t like anyone at all to know about my personal issues unless I can really trust the person so I try to limit the number of people I share my health problems with.

*(Kwesi, 35 years, Amoam-Achiase, rural)*

The second phase of contact consisted of persons who were locally acclaimed as knowledgeable about health issues including quasi-health personnel. These were people who were usually outside of close social domains of participants. Such persons included ‘educated’ persons or those who for many reasons—including serial contacts with health personnel and even years of experience using particular health service had become conversant with basic health practices and information. The third phase of contact consisted of local leaders, relatives, and friends in urban or distanced communities and health personnel (if there was any in a village). Contacts with these individuals often reflected a situation that had escalated or had the possibility of escalating into a serious one. Local leaders and other opinion leaders were considered as people with first-hand information and even experience with health-related challenges and were used for ‘privileged’ information. They were also consulted when a means of transportation (commercial car) was needed to get the sick to a medical facility.

When someone falls critically ill, and there’s no car, or it happens at night, …We usually talk to the assemblyman [local political representative]. He’s the one who can mobilise for support easily. … When there’s no car or if there’s a problem in the night, the young men carry the sick person to the nearest health facility.

*(Abena, 41 years, Afrancho, rural)*

I have made arrangements with the drivers at Foase for a car to come over every day. I have the phone number of some of the drivers. …I call them during medical emergencies if there is no car or it is late.

*(Local political leader 2, rural)*

Relatives and friends in urban and distanced communities were sometimes consulted for more health information, financial support and even in deciding the best course of action about an impending or existing health issue. The next category of network consisted primarily of health personnel. The commonest health personnel found in many rural areas were semi-trained pharmacists. In communities with health centres or clinics, health personnel (usually nurses and midwives) were consulted at this stage. Severe health conditions or urgent need for health information were rushed directly to a trained health professional or even a health service facility (the final stage). Usually, transitioning from the first stage to the second, third and fourth stages was either through direct contacts or referral by individuals/groups within the cohort of the previous set of contacts. Figure 1 sums up the processes involved in making decisions and actions about healthcare access among rural inhabitants diagrammatically. 

(b) Urban settings

Across urban spaces, members of the nuclear families, close friends and housemates were usually the first points of call for various health-related information and assistance:

I usually discuss health matters with my husband… Apart from him I often discuss such issues with my friend. …She lives in this house too. …But if I’m feeling sick I usually consult the woman here [a pharmacy attendant]. …If her prescriptions do not help then I go to the hospital.

*(Christy, 37 years, Nhyieso, urban)*

*I’ve realised that people only come here when their condition has worsened. …I think people don’t really care about their health. …Sometimes, they only come here when their condition is worse. …People usually self-medicate instead of coming to us. Many people take leftover drugs from their family members and neighbours for any symptom of illness.*

*(Medical officer 2, urban)*

Given the abundance of different kinds of health personnel in urban settings, residents casually contacted pharmacists, nurses and even doctors, and other allied health personnel for health information and directives as their second point of call. The third point of contact for many urban residents was distanced family members and group fellows. Unlike rural dwellers, urban residents relied more on their friends and housemates for everyday needs as opposed to reliance on extended family members. Indeed, some urbanites had their housemates as their closest friends. Extended family members were mostly alerted about health issues for emotional and moral support although some frequently consulted their distanced relatives for specific health information and financial assistance. The fourth stage, just like the rural folks consisted of health facilities. However, some participants were hesitant to use this option right away for financial reasons:

These days the health insurance [the National Health Insurance Scheme) do not cover many treatments. …I cannot rush to the hospital for every problem. I can only do that for my child. …Healthcare is very expensive now, so I always try to exhaust all my options before going to the hospital.

*(Addy, 48 years, Oforikrom, urban)*

Despite such inherent attitudes about reliance on social networks, cases adjudged as ‘serious’ or complicated were reported hurriedly to a health facility, just like among rural dwellers. The urbanites’ use of health formal facilities/services as their first point of call for curative, preventive and health-related information and its application was comparatively frequent than rural dwellers. This difference is depicted by the thicker direct arrow in Figure 2 compared to that of Figure 1. The width of the direct connection (arrow) between individuals and health facilities/services demonstrates the intensity of interaction between individuals and the health facilities in Figure 2. The two heuristic frameworks demonstrate the difference between rural and urban residents in how they activated and depended on different social networks to access healthcare. It is worth noting that contacts with the second and third and even the fourth network cohorts sometimes depended on the recommendations of the preceding ones as indicated by the broken arrows in both figures (interactive contact).

The diagrams depict the quintessential networks and the interactions between these networks in health-related decisions. Moreover, it was evident from the two figures and the preceding experiences of the participants that both rural and urban residents valued their proximity to the person or organisation as part of their decision making. This is demonstrated in how both groups relied on family ties and housemates as one of the first points of call.

## 4. Discussion

The study explored nuances of how known determinants of health (social networks and access to healthcare) interact in their roles, and precisely the systematic ways in rural and urban dwellers activate social networks for purposes of healthcare access. Consistent with previous works, reliance on social networks for matters of healthcare was common. Among both rural and urban dwellers, it was something which was deliberatively carved [21,28]. Rural dwellers relied on their networks for financial support not only to pay for health services but to also cater for indirect expenses such as transportation. These observations are similar to happenings in comparable and dissimilar contexts where rural residents have been found to carry a double-burden of barriers (direct and indirect) to healthcare [6,48]. Although urban residents had abundant sources of health services, financial barriers remained a hindrance to their access. The situation is imputable to weaknesses in the healthcare financing system which has seen noticeable depreciation in the past few years leading to increased out-of-pocket expenditure [9,18]. Writing on the role of social networks in western countries, Rostila [49] argues that social networks sometimes serve as a safety net for especially the lower and middle class in societies with weak welfare systems. However, it was surprising to observe that emotional support was valued in matters of healthcare use. Such revelation explains why some have argued that one’s social networks can alter demand for and efficacy of health services [25]. Intangible support from both intimate and weak social relations are known to motivate people towards greater heights even in times of distress. In matters of health, it reassures the sick of their worth and a sense of hope [17,29,31]. Studies indicate that emotional support sometimes triggers other forms of resources held in one’s social networks such as information and instrumental support [17].

The findings support the assertion that social networks of individuals can at least be described or even be independently compared across groups—rural and urban residents in this study—in terms of social processes [40]. To a greater extent, the premise of the SOS framework holds true given how the participants systematically involved their relatives, friends and other social institutions in their decisions about healthcare [38]. Indeed, in relation to health and well-being, the SOS posits that “people generally neither make a single choice nor plan a set of choices; they continue to ask advice and seek help from a wide variety of lay, professional, and semi-professional others until the situation is resolved or options are exhausted” [38]. This was particularly true for the rural residents in the present study who for various difficulties in accessing health services including health information, consistently depended on their close social networks to make decisions and sought resources to do well by their health. Moreover, according to the findings, the differences between rural and urban inhabitants concerning the individuals and groups considered as essential to health-related decisions can be attributed to some factors. Among these include issues of proximity, cultural practices and expectations, perceived knowledge of a person, and depth of financial resources embedded in the given relationship. Others comprise perceived trustworthiness and sense of fairness (including respect for one’s privacy) of individuals and institutions involved as observed in previous studies [7,11,12,23,29].

The meaning and attitudes culturally ascribed to some health conditions may have contributed to the selection of individuals and groups for consultation on health matters at the household level. Some myths and misconceptions and sometimes stigma attached to certain health conditions could have dictated the kind of persons many consulted or confided about health matters. For instance, in many sub-Saharan African settings, health conditions such as mental illness and HIV/AIDS are highly stigmatised [50,51,52]. Both sufferers and their families suffer the stigma. Hence, families and other relations of the sick sometimes remain discreet regarding such ailments. One can argue that stigma and culturally induced misinformation determined the calibre of people that participants consulted. Tenkorang and Owusu [53] blame such myths and misconceptions and the ensuing stigma on low levels of knowledge and educational attainment, which is often stark among rural dwellers. This partly explains why the incidence of health problems was kept in tight-knit social relations at the onset. Nonetheless, as the findings indicated, some social networks were sometimes too critical to ignore or kept in only close circles regardless of the stigma, and how discreet one would wish to be. For instance, financial challenges inevitably diffused notions of stigma and hesitations attached to some health conditions/information among both rural and urban people. The willingness to break such sociocultural boundaries to exploit support offered by different forms of social networks give credence to the vitality of the phenomenon in the study area [54,55]. 

From the heuristic frameworks and the views of participants, it was apparent that abstract elements such as trust and sense of fairness accounted for a significant part in decisions about the categories of individuals and groups of people choose to consult in times of adversity (or otherwise). Such cognitive elements had more to do with perceived knowledge of a person or a group and possession of resources in relation to an issue at hand and within the cultural sphere, whether a person could be trusted to remain discreet. From the participants’ accounts, such abstract elements partly explain why even husbands would not discuss solutions to their ill health or health problems with their wives but their friends and brothers. Moreover, these actions demonstrate how people segment their social networks based on perceived resources and competence, regardless of the degree of intimacy. Furthermore, this knowledge helps to disentangle how social networks are activated in terms of social depth in the decision-making process in line with tenets of the SOS framework. Thus, merely informing a person about an issue did not entirely capture the essence of activating social networks as the literature may seem to portray [38,40,41]. Aside from that, the essence of these non-physical elements in social networks may explain why previous research among non-indigenous groups—a phenomenon which is common in urban settings, observed that having strong social ties reduced the time between needing healthcare and actually making a visit by 30% due to efficient information dissemination [27].

Nevertheless, among both urban and rural dwellers, it appeared that physical proximity defined the nature of social network activation significantly. This explains why rural dwellers relied more on their extended family members than urban residents. Thus, ease of access shapes the immediacy with which some aspects of social networks are activated. The essence of physical proximity to network activation among rural residents may be enhanced by increasing access to digital technology despite numerous challenges. However, people are likely to prioritise and activate social networks that are easily accessible—in geographic terms—even if one has access to digital technology. This is why urban residents considered their extended families only at the third phase despite their comparative advantage in access to telecommunication technology [35,56]. Indeed, studies show that even availability of virtual and media infrastructure do not exclude the preference for face-to-face encounters [12]. From a sociological perspective, the proximity to social networks portrays a sense of community, promotes trust, and elicits a sense of fairness, which makes people forthcoming about their problems to varying individuals and even communities within their social networks. Consistent with the SOS framework, the findings suggest that health outcomes and health system itself are generated through continual consultation between laymen (family and friends, co-workers, classmates, neighbours), and quasi-specialists and specialists (medical doctors, traditional healers, administrators) or as concerted acts among laymen to cure, alleviate or and even educate about health-related well-being [38]. 

An apparent difference between the two groups was also the frequency and density of contact with professionally trained health personnel and the health system. These differences could be attributed to two factors. First, rural dwellers are historically disadvantaged regarding access to healthcare, particularly health facilities and personnel [9]. As such, contacts with health personnel for purposes of health education and even informal health services were a challenge. Second, rural dwellers, due to low access to healthcare often rely on informal and unapproved alternative treatments with the help of their social networks, which explains their low reliance on formal medical practitioners [13]. Extensive dependence on this approach sometimes derails effectiveness of measures geared at improving access to healthcare and results in poor health-related quality of life [57]. 

### Limitations

While the study expands knowledge on social networks and access to healthcare, it is essential to consider its limitations. The diagrammatic representations of network activation by rural and urban residents could only be temporal in the sense that the factors that necessitate specific networks change with time and are predicated on prevailing conditions [12,36]. Also, the heuristic models could have had a lot more interactions (as shown by the arrows) as the realities of human engagements are more complicated than shown here. Furthermore, the paper could not account in detail for individual characteristics of participants such as age, gender, economic status and educational attainment, which are known to affect the nature and extent of influence of social networks [20]. Nevertheless, the models provide some evidence of the complexity of how social networks are operationally adopted for purposes of healthcare access. The paper contributes to a contextual understanding of network characteristics and patterns between individuals and network actors of the two groups. 

## 5. Conclusions

The study applied the SOS framework to analyse decisions and actions about access to healthcare among rural and urban populations in the Ashanti Region in Ghana. The findings present social networks as crucial as other mechanisms for improving access to healthcare. Activation of different aspects of social networks among rural and urban dwellers to enhance chances of healthcare access is predicated on the extent of proximity, trust and sense of fairness, privacy, perceived knowledge and other resources (mainly money) that may be held in some networks. These factors shape the priority given to different individuals and organisations in one’s networks. However, the difference in network categories between the two groups—considering these factors— implies that people who are disadvantaged (such as rural dwellers) by virtue of the kind of individuals and organisations in their immediate networks will continuously remain deprived and may compound their need for multiple networks to ascertain adequate healthcare. Hence, social network analysis can pave the way for identifying and understanding the hinderances of vulnerable groups concerning healthcare access. Consequently, a critical analysis of social networks may help to tailor policy contents to the diverse needs of individuals and groups of varying socioeconomic conditions and locations.
